# The Evaluation of Rural Outdoor Dining Environment from Consumer Perspective

**DOI:** 10.3390/ijerph192113767

**Published:** 2022-10-23

**Authors:** Mian Yang, Wenjie Fan, Jian Qiu, Sining Zhang, Jinting Li

**Affiliations:** 1 Faculty of Architecture, Southwest Jiaotong University, Chengdu 610000, China; 2 Faculty of Art, Sichuan Tourism University, Chengdu 610000, China

**Keywords:** social media data, outdoor dining environment preference, rural restaurants, human-oriented, rural sustainable development

## Abstract

The quality of the environment should be measured by the satisfaction of the public and guided by the issues of public concern. With the development of the internet, social media as the main platform for people to exchange information has become a data source for planning and management analysis. Nowadays, the rural catering industry is becoming increasingly competitive, especially after the pandemic. How to further enhance the competitiveness of the rural catering industry has become a hot topic in the industry. From the perspective of consumers, we explored consumers’ preferences in a rural outdoor dining environment through social media data. The research analyzed the social media data through manual collection and object detection, divided the landscape of the rural outdoor dining environment into eight categories with 35 landscape elements, and then used BP (Back Propagation) neural network nonlinear fitting and least square linear fitting to analyze the 11,410 effective review pictures from eight rural restaurants’ social media comments in Chengdu. We derived the degree of consumer preference for the landscape quality of the rural outdoor dining environment and analyzed the differences in preference among three different groups (regular customers, customers with children, and customers with the elderly). The study found that agricultural resources are an important factor in the competitiveness of rural restaurant environments; that children’s emotions when using activity facilities can positively influence consumers’ dining experiences; that safety and hygiene environment are important factors influencing the decisions of parent–child dining; and that older people are more interested in outdoor nature, etc. The research results provide suggestions and knowledge for rural restaurant managers and designers through human-oriented needs from the perspective of consumers, and clarify the preferences and expectations of different consumer groups for rural restaurant landscapes while achieving the goal of rural landscape protection.

## 1. Introduction

The catering industry can become the core of destination development, which, in turn, can promote overall economic development to achieve the goal of sustainable development [1]. As an important development strategy put forward by China in recent years, the Rural Revitalization Strategy puts forward higher target requirements for the construction of agriculture, farming, and rural areas. Rural tourism, as an important pathway to rural revitalization, is highly valued at this stage. At the same time, the field of rural tourism research requires more comprehensive research from a wider range of academic disciplines [2]. As an important part of rural tourism, rural catering has ushered in a historic development period under the good momentum of rural tourism development [3]. The rural catering industry is vital to promoting the rural economy and protecting local cultural capital, and can also promote the joint development of urban and rural areas. As an important attraction for rural restaurants, the rural outdoor dining environment (ODE) plays an important role in the development of rural restaurants [4]. As a traditional settlement, the countryside is an important spatial carrier for agricultural production, ecological conservation, and cultural inheritance, and it has irreplaceable functions and status as urban areas [5]. The harmonious integration of “bottom-up” human-oriented needs can enhance the freedom, continuity, and diversity of the development of individual forms of villages, and avoid the “one size fits all” development model of villages caused by planning assumptions lacking human-oriented considerations [6]. Therefore, we believe that the research of rural ODE focusing on consumer needs is very important for rural revitalization and rural sustainable development.

### 1.1. Rural Outdoor Dining Environment

With the increased interest of urban residents in rural areas, the rural landscape has promoted the development of rural tourism [7] and highlighted its economic value [8]. The properties, leisure infrastructure, culture, and natural landscape of rural tourism sites are all important pull factors for rural tourism [9]. Under scientific tourism development, these resources can be better protected and sustainable development of rural resources can be realized [10]. Food tourism has a very important role in rural tourism, which creates more jobs for local people and promotes economic development [11,12]. A study by Scozzafava et al. [13] found that restaurants that supported local food and organic products positively influenced customers, and that restaurants with local products were three times more likely to be chosen than restaurants without local products. Rinaldi et al. [14] studied the local identity and attractiveness of rural areas and agriculture, and suggested that local dining resources must address and strengthen the link between place (territorial/geographic dimension) and people (cultural dimension). Food, the environment, and novelty value are the main prerequisites for attracting consumers to promote urban and rural co-development [15,16]. It can be seen that the study of the rural dining environment contributes to the development of rural tourism as well as the sustainable development of the rural landscape.

Customers need a unique dining environment to enjoy a different experience (Liu and Jang, 2009) [17]. With the change in lifestyle, dining out in rural areas has become an important social behavior. Auty (2006) found that whereas consumers stated that food type and food quality were the main variables in choosing a restaurant, as consumers’ dining needs increased, the environment of the restaurant became the determining factor [18]. Therefore, the physical environment of catering is very important in shaping the image of restaurants and influencing customer behavior [19,20]. Scholars have also studied the relationship between consumers’ dining experiences and individual factors. Based on quantitative analysis, Ryu et al. identified a six-factor scale consisting of facility aesthetics, ambiance, lighting, service offerings, layout, and social factors as a procedure to assess DINESCAPE in upscale restaurant environments [21], and found that facility aesthetics, atmosphere, and staff had a significant impact on customer pleasure [22]. Hong and Hsu [23] summarized restaurant interior environments into four dimensions: physical environment (architecture, restaurant name, sign, interior design and decoration, furniture and equipment, layout, lighting, temperature, aroma, and music), product and service (appearance and flavor of food and beverages, plating, the items on and design of the menu, tableware, employees’ expressions, employees’ physical movement and gestures, employees’ introductions, communication, and storytelling), employee’s aesthetic traits (appearance, voice, and body odor), and other customers’ aesthetic traits (customer appearance, voice, behavior, and etiquette). Yang et al. [4] proposed three ODE dimensions that influence consumer satisfaction with rural restaurants: quality and facilities (uniform, appearance, garnish, table setting, service quality, table placement, illumination, and decorations), image and atmosphere (name, natural sound, signage, and music), and landscape elements (pavement, artificial structure, buildings, and ornamental plants), and found that customers in rural areas tend to prefer to experience natural landscapes, and no other study proposed the ODE dimensions as far as we know. Albright et al. [24] found that women and older adults tend to be more interested in making healthy choices in restaurants. Bai et al. [25] also found that women were more selective in choosing safe restaurant environments to protect themselves. Based on the above, we know that most of the previous studies on the dining environment focused on the building or indoor environment, and few studies focused on the consumer preference of ODE in rural restaurants [4]. The outdoor environment of a rural restaurant differs somewhat from the influencing elements of the indoor environment, and together they affect consumer satisfaction in dining. The outdoor and indoor environment elements, the requirements of different customer groups, and consumer preference for the rural landscape elements of rural restaurants all have some differences, and together affect consumer satisfaction [26]. Therefore, the improvement of the rural ODE is very important to enhance the attractiveness of rural restaurants.

Ayala et al. [27] called for a more microscopic and nuanced look at the interactions of participants in order to understand the interactions of multiple stakeholders in urban construction and the conflicts and risks that arise from them. However, to the best of our knowledge, there is little support for the study of rural ODEs and the refinement of dining environments. This helps to advance the creation of a landscape environment for rural restaurants, thus achieving an improved ODE for rural restaurants. In summary, the refined classification of consumer preferences for rural outdoor dining environments has significant value and can provide theoretical help and advice to restaurant managers and planners in various aspects of planning, design, and management. Therefore, we proposed, for the first time, a refined study of consumer preferences for the quality of the rural ODE from the perspective of social media user-generated content, and argued that the results can help the rural catering industry to improve its competitiveness and the sustainable development of rural areas.

### 1.2. The Use of Social Media Data in Landscapes

Big data has now shown scientific advantages in tourism research. Humanism and data application will be the two major themes of future urban development [28]. When human behavior and social activities are deeply data-driven, human needs can also be finely measured and predictively analyzed [29]. In recent years, social media user content and other data in urban planning and landscape design have also provided a substantial scientific basis for the study of users’ aesthetic preferences, perceptions, activity patterns, and other issues. Guan et al. [30] found significant seasonal variation in park visitation through anonymous phone location data and review content from local review sites, and seasonal fluctuations in park spatial characteristics in relation to seasonal activities, visitor perceptions, and visitation patterns. Using social media photos from Flickr and Panoramio, Tieskens et al. [31] estimated correlations between landscape attributes and landscape preferences, arguing that social media data can serve as evidence of the value of landscape elements, the location of people’s interactions with the landscape, and how these interactions characterize the landscape. Li et al. [32] combined visitor ratings obtained from social media with government assessment scores to study visitor preferences for cultural ecosystem services in rural landscapes. Natural landscapes, infrastructure, and services were found to have a significant impact on the public in rural landscapes, and the relationship between different rural landscape features was not consistent across preferences for cultural ecosystem services. The findings enrich the dimension of sensory elements of cultural ecosystems and better support the management, planning, and conservation of rural landscapes. Zhang et al. [33] conducted a thorough complexity, visual scale, and color study of the visual attributes of the landscape for each attraction by evaluating photos posted by Sina Weibo users, based on a fixed-point photography experiment. The mapping relationship between the visual attributes of the landscape space and the perception of the observer was revealed. Huang et al. [34] presented a study on the application of big data in improving landscape plant gardening methods and found that the metrics of big data landscape design outperformed traditional landscape design. Several studies have shown that social media data play an important role in the field of landscape research, even complementing traditional data analysis methods, and exploring the role of landscape elements in management, planning, and conservation from research perspectives such as landscape preference and landscape perception.

Research on restaurant environment evaluation and satisfaction through social media data is also growing in popularity. Qin et al. [35] evaluated the development of the quality of the urban restaurant space at the macro level of the urban environment by using consumer review data in Dianping. Through the quantification of social media data, they found a method that can evaluate a service quality of the restaurant. Jung et al. [36] studied the changes in satisfaction with dining out before and after the pandemic through the content of comments on social media data and concluded that the study of changes in consumer dining needs is an important way to help restaurant companies adapt to the development of social changes and promote sustainable management. Koufie et al. [37] discuss what millennials look for in restaurant reviews and the importance of a restaurant’s online word-of-mouth among today’s millennial population, and also emphasize that social media should be incorporated into the restaurant’s marketing communication strategy in restaurant management. It is evident that the study of user-generated content on social media has important value for restaurant management.

Public policy research should be an integrated innovation based on a human-oriented approach, with interdisciplinary knowledge applied to the areas underlying the assumptions of rational managers [38]. Analysis of the rural outdoor dining environment based on social media data can grasp a large amount of information on consumers’ perceptions and feelings [39]. When the information is closely linked, we can provide theoretical support for tourism planning and management from the field of landscape architecture. From the perspective of user-generated content on social media platforms, this paper applies artificial intelligence to identify the sensory perceptions and their associated elements present in user-posted comments through object detection techniques in computer vision. Based on the analysis of the results, to determine consumers’ landscape preferences for ODE, to study the impact of landscape quality on consumer decision-making and dining experience in a human-oriented manner. The research purposes of this paper are as follows:

1. Study consumer preferences for the landscape environment of rural ODEs through social media user-generated content.
2. To explore which type of landscape in rural ODEs is most preferred by consumers to improve the quality of rural tourism services.
3. Provide suggestions for the construction of rural ODEs to promote the integrated development of rural culture and tourism, protect rural landscapes, and upgrade the quality of rural tourism.

## 2. Materials and Methods

### 2.1. Data Collection

Dianping is the most widely used restaurant search platform in China, providing consumers with information on restaurants, finding dining destinations, sharing dining experiences, making dining plans, and so on. The content shared by users included ratings, text comments, and images of the restaurant, which contained a great deal of information about the environment, such as seating, decor, and service, and served as the main source of data collection for our experiment. As a city with a tourism orientation of “tourism destination dominated by Chinese rural vacation”, Chengdu is the origin of Chinese agritainment and the representative of rural tourism development in China. Outdoor dining and recreation are one of the key features of Chinese agritainment tourism. We chose the Taohuaguli scenic area as the research site. It is a famous rural tourist attraction in Chengdu with a focus on gastronomy tourism and agricultural sightseeing, and is known as a “National Famous Town for Special Landscape Tourism”. In 2021, the village received 1.83 million tourists and had a tourism income of nearly CNY 200 million [40]. Based on consumer ratings, we have selected the eight highest rated country restaurants in the area for our data collection: Shouhuangjiang (tea and gastronomy, 1288 images), Longquan Banshan Villa (B&Bs and restaurants, 1515 images), Yunlixiaozuo (Sichuan cuisine, 3927 images), Liangwang (B&Bs and gastronomy, 1490 images), Mo’antaoli (afternoon tea and private kitchen, 1602 images), Dengxian (Sichuan cuisine, 511 images), Creeper (B&Bs and gastronomy, 288 images), and Picnics restaurant (gastronomy, 789 images).

We collect user-generated review images from the rural restaurants through Dianping and use object detection, a statistical method for detecting a certain class of semantic objects in digital images, to analyze and extract statistical information from images instead of manual labor. Object detection is a research hotspot in computer vision and has been widely used in social science research [41]. A total of 11,583 images were obtained, of which 173 sample images were excluded because they were too blurred to identify the landscape elements. A total of 11,410 valid sample images were obtained, with a validity rate of 98.5%.

### 2.2. Data Classification

Interventionary studies involving animals or humans, and other studies that require ethical approval, must list the authority that provided approval and the corresponding ethical approval code.

In the process of sorting out the image information, we combined the three dimensions proposed by Yang et al. [4] with the ODE landscape elements that frequently appear in the statistics for preliminary classification, and classified the elements into 37 types. Then, we referred to the Delphi method [42] that Bao et al. used to classify wetland landscapes [43]. Three experts (including a professor of rural landscape planning research, an experienced rural landscape planner, and a rural restaurant manager) were invited to conduct a field survey. Considering the functional characteristics and landscape features of each landscape element, the final classification was into eight broad categories and 35 specific landscape elements (Table 1).

To further clarify the target differences in market segments, we classified posts with the keywords “parents, elders” as the elderly dinner group, with 2704 photos screened, and classified the comments with the keywords “children, kids, teenagers” as the parent–child dinner group, with 4141 photos screened, and the overlap between the two groups was double counted and 1323 photos were screened.

### 2.3. Data Processing

**Figure 1:**
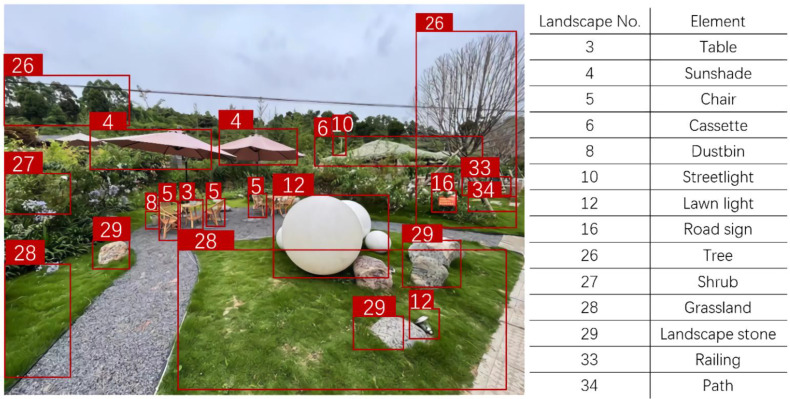
Object detection model data recognition diagram.

The object detection algorithm has been widely used in the field of image recognition [44,45]. In this research, the Yolo algorithm [46] is used to supervise the object detection network, and a sample set of 35 landscape elements was constructed from the dataset, each containing 20 pictures, a total of 700 images were used to train the object detection model to learn to recognize various landscape elements. We then fed 11,410 images of the rural ODE evaluation comments into the object detection model and counted the elemental information contained in each image, as shown in Figure 1.

**Figure 2:**
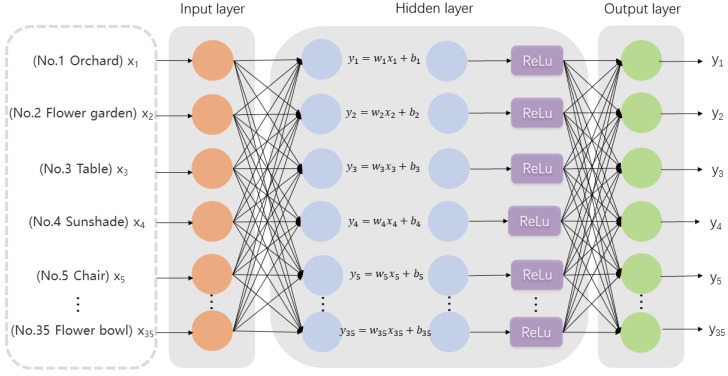
The BP network model.

The BP neural network model is a multilayer feedforward neural network model trained according to the error backpropagation algorithm with arbitrarily complex pattern classification capability and an excellent ability to map multidimensional functions and fit nonlinear models. The neural network consists of an input layer, an output layer, and a hidden layer with a custom number of layers, which consists of a number of neurons. In the forward transmission process of the BP neural network, the neuron in the latter layer receives the input signals transmitted by the neuron in the previous layer and assigns weights to these signals. The summation result is compared with the threshold value of the current neuron, and then the result is processed by the activation function to obtain the output score [47]. Due to the large amount of data, we chose the ReLu activation function in order to reduce the dependence between parameters, reduce the overfitting rate, and enhance the robustness of the model. The output result as Formula (1):

Yi = ReLu (WiXi + b) (1)

where Xi is the input value, i.e., 35 elements, 1 for presence and 0 for absence; Wi is the weight and Yi is the output value. The BP network structure is shown in Figure 2.

**Figure 3:**
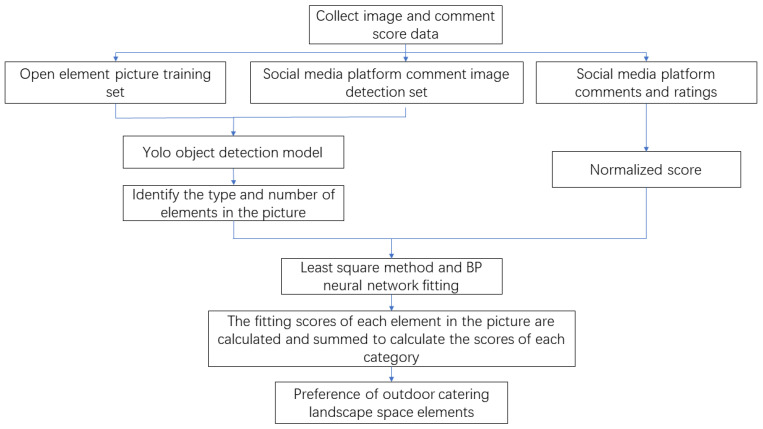
Research framework.

In the model training process, we extracted 80% of the sample set for BP neural network training, which is used to construct the relationship between landscape elements and restaurant ratings. A total of 20% of the sample set was used for the test set, which is used to verify the effect of the training model. A cross-validation method is used, whereby the training and test sets are randomly divided and averaged over multiple training sessions. The output value of each element is the landscape element score, thus comparing and analyzing consumers’ landscape element preferences in the rural ODE, and the research framework is shown in Figure 3.

### 2.4. Validation of the Fitting Effect

**Figure 4:**
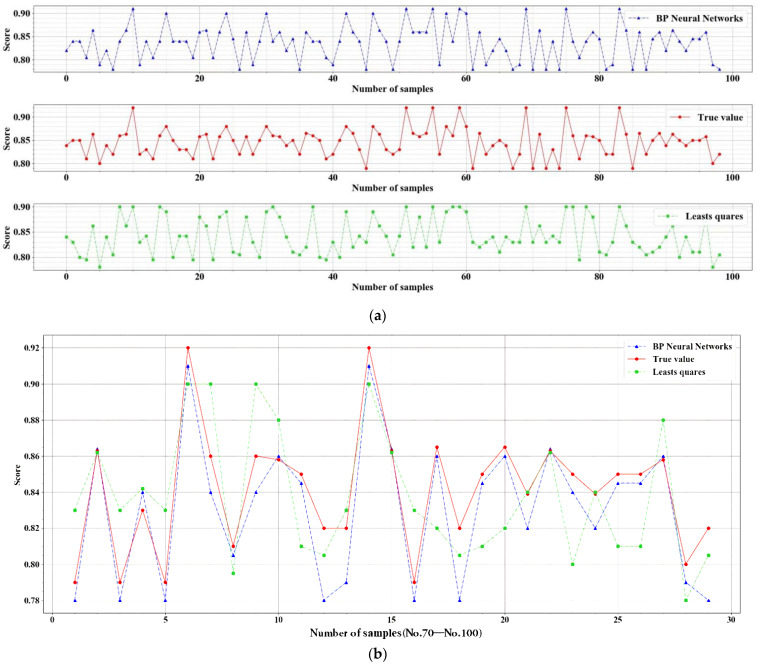
(**a**) shows the fitting result under each methods of a 100 samples cut; (**b**) shows the comparison results of fit rates of a 30 samples cut.

The least square method is used for linear fitting, and the BP neural network is used for nonlinear fitting to verify the accuracy of model fitting. Normalize the score so that its range is in the interval [0,1]. Define the ratio of the number of samples with a relative error of ± 0.1 to the number of all samples as the fitting rate as a measure of fitting index. The fitting rate of the BP neural network is 89.579%, while the fitting rate of the least square method is 87.564% (see Figure 4). Although both fitting methods are effective, the BP neural network model has considered certain nonlinear factors, and the generalization effect of the model is better. By extending the batch processing with BP neural networks, adding a regularization module, and setting a small learning rate, the fitting results can be prevented from affecting non-significant data [48], the research findings can be more objective and valid. Therefore, we choose the fitting results of the BP neural network for discussion.

## 3. Results

### 3.1. Overall Fitting Results

The fitting results of landscape categories and elements are shown in Table 2. From Table 2, the preference ranking of the eight categories of the consumers is children’s facilities (0.8740) > service (0.8703) > landscape (0.8670) > lighting (0.8593) > recreation facilities (0.8475) > sanitary facilities (0.8393) > production landscape (0.8275) > guided tour (0.8237).

From the preference of landscape elements, we find that slides and seesaws are highly preferred across all. Natural landscapes, such as plant landscapes and the view of the scenery, and artificial landscapes, such as feature walls, sculptures, and flower bowls, are all highly preferred. The preferences of catering decoration, cassette, flower garden, and art board are significantly higher among similar landscapes, while the preference of orchards, railings, and billboards are the opposite.

### 3.2. Fitting Results of Parent-Child Group

From Table 3, the preference ranking of the eight categories of the parent–child dinner group is children’s facilities (0.8985) > sanitary facilities (0.8706) > lighting (0.8567) > recreation facilities (0.8560) > landscape (0.8531) > production landscape (0.8525) > service (0.8473) > guided tour (0.8170).

In detail, we find that all the children’s facilities, such as slides, seesaws, sandpits, and swings, are highly preferred. Clean sanitary facilities with the preference of washbasins and toilets are highly preferred. Natural landscapes, such as plant landscapes, and artificial landscapes, such as feature walls, sculptures, and railings, are highly preferred. The preference for chairs and sunshades is significantly higher in the recreation facilities. The preference for light strips, flower gardens, and attendant dress code is higher among the similar landscape elements, while the preference for road signs, art boards, and waterscapes is the opposite among similar landscapes.

### 3.3. Fitting Results of Elder Group

From Table 4, the preference ranking of the eight categories of the elderly dining crowd is from high to low: recreation facilities (0.8840) > landscape (0.8781) > service (0.8680) > production landscape (0.8520) > sanitary facilities (0.8516) > guided tour (0.8213) > children’s facilities (0.8090) > lighting (0.8067).

From the preference of landscape elements, we find that chairs, cassettes, sunshades, and tables are highly noted in this group. Natural landscapes, such as plant landscapes and views of the scenery, as well as artificial landscapes, such as feature walls, railings, flower bowls, and landscape stones, are highly preferred. The preference of catering decorations, flower gardens, dustbins, street lights, and road signs is significantly higher among similar landscapes, while the opposite is true for lawn lights, sandpits, and spotlights.

## 4. Discussion

Rural products with a high degree of localization are the basis for the development of rural tourism. The traditional rural landscape is highly distinctive and can enhance the local tourism brand [49,50]. With eight types of landscape categories and 35 landscape elements summarized through the statistics of 11,410 photos collected from social media data, this is the first study to refine the classification of rural ODEs and is an important contribution to this field of study. We used BP neural network analysis to score the preferences of the landscape types and the landscape elements. In terms of theoretical research, we studied consumers’ experiential preferences in rural outdoor dining environments, explored the use of rural landscape elements in rural restaurants, and called for the reasonable protection and utilization of rural resources with positive values. In practice, this paper suggests that planners and managers can take advantage of the localization and seasonal variation of production landscapes, clarify the target positioning of restaurant clientele, and complete the construction of infrastructure services for different groups, including protection of rural resources and the environment (biodiversity and natural and human landscape resources) to promote the competitiveness of rural restaurants and to improve the competitiveness of rural restaurants, while contributing to sustainable rural development.

Different people have different needs and preferences for ODEs of rural restaurants, and understanding the behavioral needs of different groups of people when it comes to outdoor recreation is becoming increasingly important [51]. In terms of overall consumer preference score, consumer preference for children’s facilities is significant. When children regard an activity as a game, they show more signs of emotional health [52], and the pleasure soundscape and multitasking of children’s play have a positive impact [53]. Therefore, we concluded through our study that the emotional perceptions displayed by children when using the children’s facilities in rural ODEs can have a significant positive impact on consumers’ dining decisions, yet children’s activities are often ignored in research as an element of restaurant attraction. Agriculture and production, as important cultural and natural resources in the traditional countryside, are important pull factors for rural tourism and also influence consumers’ preference for rural restaurants. In its landscape planning, rural restaurants should pay attention to both the functional expression of the landscape in terms of cultural, historical, educational, and research values of the agricultural landscape; otherwise consumers will be limited in the experiences they receive and the ways they can participate [54]. The agricultural landscape is the main component of agricultural culture, and they are mutually reinforcing. Rational use of agricultural resources around a rural restaurant is a win-win model for both the restaurant and the rural area. The preference for the seasonal characteristics of agricultural landscapes in the dining experience of rural restaurant consumers and the value of agricultural culture in rural restaurants are also worthy of further study. In this study, the landscape category shows a high preference, and in the planning and design process, more refined design considerations should be made for its specific elements, such as Rossetti et al. [55] suggesting that railings have a positive impact on aesthetics and safety, but they are easy to ignore and lead to inactivity and boredom. Even in our study, as an important landscape element, Zhang et al. [56,57] believe that walls have a comprehensive negative impact, even causing depression and boredom, because they hinder the green landscape or accumulate pollutants. It is clear that functional infrastructure can have a positive impact and appeal if it is well designed. Therefore, in the design of infrastructure, it should also be integrated into local culture for careful design consideration.

In contrast to the needs of different groups of people, children’s facilities can meet the needs of interaction between parents and children [58], but also influence the consumption decisions of the family dinner crowd [59]. This study argues for this result, proving that children’s activities have a positive impact on consumers of outdoor dining in the rural area, and also finding in the published user-generated data that four types of children’s landscape facilities—sandpits, seesaws, slides, and swings—are highly attractive to the parent–child gathering group. The choice of colors, materials and types of children’s facilities needs to be studied more finely in relation to different environments and specific groups [60]. This study complements the results of the selection of facility types in a rural ODE. The sanitary environment is also a high concern for family dinner groups in the outdoor environment of country restaurants, especially after the outbreak of the COVID-19 pandemic [61]. Although evolutionary theories of landscape preference suggest that people naturally prefer waterscapes [62], and that the presence of water triggers preference and pleasure, and that it always enhances visual quality [63], in the parent–child group, we found that consumers preferred water features less than other elements. Meanwhile, the parent–child group also has a low preference for environments that are dangerous to children, such as viewing platforms and paths that may blur the borders. Instead, there is a greater preference for elements, such as railings and feature walls, that have a separating and enclosing effect. Therefore, we believe that safety and hygiene are important factors that influence the decision of the parent–child group to ODEs in rural restaurants. The kinds of children’s play spaces in a rural ODE that can better allay parents’ concerns about safety in order to improve consumer satisfaction with the environment are subject to further research. Moreover, children are extremely sensitive to the physical environment, especially the light environment [64]. A reasonably good light environment can elicit positive emotions and a desire to explore, [65] has similar findings to our study, and we believe that suitable restaurant lighting environment has a certain appeal and competitive advantage for parent–child groups. Those dining with their elders are more interested in the leisure and landscape categories, and it can be argued that elderly people prefer to eat and relax outdoors in good environments [66]. Therefore, restaurant managers could create a leisurely and beautiful traditional agricultural landscape to make the ODE of rural restaurants more humane and naturalistic for elders to rest and enjoy the view. In addition, the group dining with elders also pays high attention to the service, and restaurant managers should pay attention to it in terms of service facilities for the elderly. Another item that stands out is the high preference for good street lighting among the group dining with elders, perhaps for safety reasons, but they have a relatively low preference for spotlights and strip lights. To sum up, there are some differences in consumer preferences between parent–child groups and dining with elder groups. Restaurants aimed at these two groups should be designed and managed with quality and hygiene, green and nature, and consumer safety as the focus of ODE in rural restaurants. In response to the different preferences between different populations suggested by the study, researchers of children’s facilities, children’s safety, and landscape lighting could also conduct further and more detailed studies in rural areas.

Combined with the results of the three groups, we find that consumers are highly interested in the landscape category. This result can be explained by the fact that consumers have a tendency to seek naturalization of the environment and a higher preference for landscapes with local characteristics, and it also confirms that “naturalness” is an important factor in landscape preference [67,68]. Historical culture and natural resources in the traditional countryside are important pull factors for rural tourism, whether it is at the planning level, design level, or management level, neither the culture nor the natural resources of the traditional countryside should be ignored in the face of its value. The conservation and sustainable use of rural landscape resources through scientific and technological means is essential for the full implementation of the principle of ecological priority. The ODE of rural restaurants can provide a natural, comfortable, and authentic environment for consumers, using the rural landscape environment with local characteristics to attract consumers to achieve the purpose of promoting the environmental protection of rural environmental resources and the sustainable development of the rural catering industry.

Urban and rural area construction and development cannot simply focus on top-down development from the engineering dimension but also need to be linked to social needs. In order to reveal the complexity of the urban and rural construction processes, the participants (subjects of interest) should be included in the scope of investigation, and a more microscopic and detailed observation of the interaction between the participants should be conducted [27,69]. With planning and management based on a human-oriented perspective, the government can reduce costs, improve effectiveness, and enhance efficiency in policy development and project design [70]. Managers can better target restaurant positioning, restaurant themes, and environmental design to improve consumer satisfaction and repurchase willingness, thereby increasing restaurant competitiveness. The research in this paper combines landscape architecture with tourism management, and through social media data analysis, we try to explore refined management of rural restaurants and human-oriented design considerations of ODEs, which can provide a reference for governments and managers. For example, Gibson et al. argue that as cities have gradually brightened up, rural landscape lighting has been lagging behind [71]. The right policies and projects may be able to effectively integrate rural restaurant lighting projects with rural landscape lighting to enhance rural infrastructure. In addition to market and social factors, restaurant managers should also consider the location of their projects in a local context, taking advantage of their location and targeting different customer segments to make a more economical, efficient, and sustainable project. Managers who need to improve the quality of their country’s restaurants can also do so by combining the most prominent problems in their restaurants with consumer preferences in order to effectively improve the attractions of their restaurants. In restaurant publicity, restaurant managers can also increase the value of advertising by targeting different groups of people according to the characteristics of the restaurant.

## 5. Conclusions

Based on the user-generated content of social media platforms, this study explores the classification of ODE of rural restaurants, analyzes consumer preferences for landscape categories and elements, and provides guidance for the development of rural tourism and rural restaurants. The results of this study can provide practical advice to planners and managers at different levels and provide some value to rural tourism development and rural environmental protection. However, this study also has some limitations: (1) The data for the study comes from social media, where most of the people active on social media are young or highly educated, and the data are not universally available. (2) In terms of segmentation, we have only divided the age groups through textual evaluation or the content of the people appearing in the photos, the results of the study can only represent the perceived preferences of consumers with children, consumers accompanying the elderly, and the general public. Further research is needed to know the preferences of specific children or the elderly themselves. (3) The classification in this study is based on an exploration of villages in western China. Villages in different regional and cultural contexts will have different landscape qualities, and future research should be conducted in different regions to test the applicability and generalizability of the landscape element preferences in this study. In addition, after the pandemic, people’s changes in the use and perception of green spaces [72], dining habits, and satisfaction are also changing [73]. Perhaps an exploration of the changes in the rural ODEs after the pandemic based on social media data will also reveal new and different findings to complement the study of consumers’ preferences and satisfaction in rural ODEs.

To our knowledge, there are few studies on consumer preferences for rural ODEs. Although we only sampled in Chengdu, China, the study is still valuable in several ways. Firstly, from the aspect of refined management and design of urban and rural planning, the study of restaurant management and design is carried out based on the real needs of people, hoping to resonate with more researchers from related fields and to jointly explore the human-oriented considerations and exploration of urban and rural planning. Secondly, the results of the study provide consumer-based recommendations for improving the competitiveness of rural restaurants in terms of design and management. At the same time, we also call on people at all levels to make rational use of the diverse values of rural culture and natural resources in order to truly achieve a win-win situation for the development and conservation of rural areas.

## Figures and Tables

**Table 1:** Classification of landscape elements of ODE.

Classification No.	Category	Landscape No.	Element	Frequency	Landscape No.	Element	Frequency
I	Production landscape	1	Orchard	1289	2	Flower garden	1380
II	Recreation facilities	3	Table	6480	4	Sunshade	4233
		5	Chair	6379	6	Cassette	5488
III	Sanitary facilities	7	Toilet	444	8	Dustbin	1563
		9	Washbasin	342			
IV	Lighting	10	Streetlight	1700	11	Light strip	1722
		12	Lawn light	4438	13	Spotlight	2293
V	Guided tour	14	Art board	1745	15	Billboard	1791
		16	Road sign	1506			
VI	Service	17	Dress code	787	18	Catering decoration	4073
		19	Catering setting	5511			
VII	Children’s facilities	20	Slide	1768	21	Swing	1905
		22	Sandpit	1243	23	Seesaw	1118
VIII	Landscape	24	Viewing platform	2430	25	Waterscape	4255
		26	Tree	8340	27	Shrub	7564
		28	Grassland	8751	29	Landscape stone	4917
		30	Rockery	2475	31	Feature wall	1585
		32	Sculpture	4415	33	Railing	4700
		34	Path	3639	35	Flower bowl	4986

**Table 2:** Overall landscape categories and elements preference.

Category	Score	Element	Score	Element	Score
Production landscape	0.8275	Orchard	0.797	Flower garden	0.858
Recreation facilities	0.8475	Table	0.836	Sunshade	0.848
		Chair	0.838	Cassette	0.868
Sanitary facilities	0.8393	Toilet	0.842	Dustbin	0.837
		Washbasin	0.839		
Lighting	0.8593	Streetlight	0.860	Light strip	0.863
		Lawn light	0.856	Spotlight	0.858
Guided tour	0.8237	Art board	0.851	Billboard	0.802
		Road sign	0.818		
Service	0.8703	Dress code	0.872	Catering decoration	0.847
		Catering setting	0.892		
Children’s facilities	0.8740	Slide	0.912	Swing	0.842
		Sandpit	0.841	Seesaw	0.901
Landscape	0.8670	Viewing platform	0.892	Waterscape	0.866
		Tree	0.881	Shrub	0.853
		Grassland	0.864	Landscape stone	0.861
		Rockery	0.878	Feature wall	0.894
		Sculpture	0.878	Railing	0.803
		Path	0.859	Flower bowl	0.875

**Table 3:** Landscape categories and elements preference of parent–child dining group.

Category	Score	Element	Score	Element	Score
Production landscape	0.8525	Orchard	0.850	Flower garden	0.855
Recreation facilities	0.8560	Table	0.833	Sunshade	0.866
		Chair	0.868	Cassette	0.857
Sanitary facilities	0.8706	Toilet	0.862	Dustbin	0.861
		Washbasin	0.889		
Lighting	0.8567	Streetlight	0.854	Light strip	0.862
		Lawn light	0.861	Spotlight	0.850
Guided tour	0.8170	Art board	0.814	Billboard	0.821
		Road sign	0.816		
Service	0.8473	Dress code	0.852	Catering decoration	0.841
		Catering setting	0.849		
Children’s facilities	0.8985	Slide	0.932	Swing	0.912
		Sandpit	0.921	Seesaw	0.929
Landscape	0.8531	Viewing platform	0.834	Waterscape	0.802
		Tree	0.861	Shrub	0.863
		Grassland	0.862	Landscape stone	0.850
		Rockery	0.853	Feature wall	0.864
		Sculpture	0.879	Railing	0.876
		Path	0.837	Flower bowl	0.857

**Table 4:** Landscape categories and elements preference of elder dining group.

Category	Score	Element	Score	Element	Score
Production landscape	0.8520	Orchard	0.837	Flower garden	0.867
Recreation facilities	0.8840	Table	0.876	Sunshade	0.883
		Chair	0.897	Cassette	0.889
Sanitary facilities	0.8516	Toilet	0.851	Dustbin	0.858
		Washbasin	0.846		
Lighting	0.8067	Streetlight	0.849	Light strip	0.814
		Lawn light	0.797	Spotlight	0.767
Guided tour	0.8213	Art board	0.819	Billboard	0.817
		Road sign	0.828		
Service	0.8680	Dress code	0.868	Catering decoration	0.861
		Catering setting	0.875		
Children’s facilities	0.8090	Slide	0.812	Swing	0.802
		Sandpit	0.791	Seesaw	0.831
Landscape	0.8781	Viewing platform	0.903	Waterscape	0.855
		Tree	0.881	Shrub	0.863
		Grassland	0.874	Landscape stone	0.871
		Rockery	0.868	Feature wall	0.889
		Sculpture	0.853	Railing	0.879
		Path	0.832	Flower bowl	0.873

## Data Availability

The data presented in this study are available on request from the corresponding author. The data are not publicly available due to privacy.
